# Morphometrical and Molecular Characterization of *Oesophagostomum columbianum* (Chabertiidae: Oesophagostominae) and *Haemonchus contortus* (Trichostrongylidae: Haemonchinae) Isolated from Goat (*Capra hircus*) in Sylhet, Bangladesh

**DOI:** 10.1155/2021/8863283

**Published:** 2021-02-24

**Authors:** Tilak Chandra Nath, Dongmin Lee, Hansol Park, Seongjun Choe, Barakaeli Abdieli Ndosi, Yeseul Kang, Mohammed Mebarek Bia, Chatanun Eamudomkarn, Uday Kumar Mohanta, Kazi Mehetazul Islam, Jamal Uddin Bhuiyan, Hyeong-Kyu Jeon, Keeseon S. Eom

**Affiliations:** ^1^Department of Parasitology, Parasite Research Center and Parasite Resource Bank, School of Medicine, Chungbuk National University, Republic of Korea; ^2^Department of Parasitology, Sylhet Agricultural University, Bangladesh; ^3^Department of Microbiology and Parasitology, Sher-e-Bangla Agricultural University, Bangladesh

## Abstract

This study was aimed at describing two (2) intestinal nematodes from naturally infected native breed of goats (*Capra hircus*) in Bangladesh, identified as *Oesophagostomum columbianum* (Curtice, 1890) Stossich 1899 and *Haemonchus contortus* (Rudolphi, 1803) Cobb, 1898. The identification was made based on morphometric features and was confirmed by amplifying internal transcribed spacer (ITS) and cytochrome *c* oxidase (*cox*1) gene. Well-developed lateral alae, distinct cervical papillae anteriorly to esophageal expansion, and male spicule length (0.73-0.79 mm, *n* = 2) were characteristically observed in *O. columbianum*. At the same time, male spicule length (0.40-0.46 mm, *n* = 2) and position of female vulvar flap (4.30-4.54 mm from posterior end, *n* = 3) were observed in *H. contortus*. DNA sequence homology of the ITS and *cox*1 gene of both specimens revealed the same results, showing similarity to the GenBank sequences of *O. columbianum* (GenBank No. KC715827; JX188470) and *H. contortus* (GenBank No. KJ724377; HQ389229). Phylogenetic analysis computed by maximum livelihood (ML) from the ITS nucleotide sequences revealed that the *O. columbianum* and *H. contortus* isolates identified in this study were clustered in the same clade with isolates from China and Iran, respectively. This study, for the first time, illustrates the characteristics of *O. columbianum* and *H. contortus* in Bangladesh, combining both morphological and molecular data. The universal primer-based polymerase chain reaction (PCR) protocol could be an economical and efficient option for researchers from poor resource settings for precise identification of nematodes. The information generated in this study may contribute to formulating effective control strategies against these nematodes.

## 1. Introduction

Infection with gastrointestinal nematodes (GINs) is considered one of the significant problems causing considerable economic losses in ruminant farming. Over 150 species of internal and external parasites have been reported to infect goats and sheep worldwide [1, 2]. *Oesophagostomum* or nodular worm is a parasitic nematode of the large intestine belonging to the family Chabertiidae (Popova, 1952) and is one of the most common and widely distributed nematodes of livestock and wild ruminants [3]. *Oesophagostomum columbianum* (Curtice, 1890), *O. asperum* (Koehler, 1930), *O. venulosum* (Rudolphi, 1809), and *O. kansuensis* (Hsiung *et* Kung, 1955) are the dominant species; in most cases, animals get infected through contaminated foods, water, or soil [2]. *Oesophagostomum columbianum* has been cited as the primary causal agent of nodular enteritis, responsible for decrease productivity in sheep and goats around the world, for instance, in Kashmir Valley, India, [2] and Ethiopia [4]. Mature worms of these species inhabit the mucosa of the host's digestive tract and suck blood that leads to pernicious anaemia and significant weight loss [1]. Penetration of the mucosa of the intestine by larvae can cause severe diarrhoea with black-green faeces containing mucus and blood. On the other hand, *Haemonchus* or barber's pole worm is a parasitic nematode belonging to the family Trichostrongylidae (Leiper, 1908; Leiper 1912) and is a blood-sucking nematode that inhabits in the abomasum of small ruminants worldwide. *Haemonchus contortus* (Rudolphi, 1803) Cobb, 1898; *H. placei* (Place, 1893) Ransom, 1911; and *H. similis* (Travassos, 1914) are reported as the most pathogenic nematodes of sheep, cattle, and goats worldwide, causing significant production losses [5]. These nematodes are found mostly in tropical regions and also reported to occur increasingly in subtropical areas [6, 7]. These nematodes live in the digestive tract of sheep and goat; adult worms suck blood from the intestinal mucosa and cause anaemia, oedema, diarrhoea, and even death [8]. Economic losses are encountered in terms of production and body weight loss, direct medication costs, and mortality-related loss.

Small ruminant, especially goat, has become an important farming system in Bangladesh for a long time. However, to date, very few studies on ruminant parasitism have been conducted for the identification of GINs in this area. While several studies reported the high prevalence of *O. columbianum* and *H. contortus* in Bangladesh from sheep and goat based on coprological approaches [6, 9, 10], explanation of key identification criteria and genetic analysis for those nematodes were lacking. The conventional coprological methods include morphology-based identification of eggs or larvae that are cumbersome and difficult to distinguish from other species with similar morphological structures or minor morphological variations. A multidisciplinary approach, including both morphological and DNA-based molecular techniques, should provide a more reliable means of identification [11]. Ribosomal DNA (rDNA) genes and their related spacer regions and mitochondrial DNA (mtDNA) provide useful information for the development of diagnostic probes or species identification makers in this regard. While molecular methods are the gold standard for the precise identification of nematode species, identification based on the morphometry is cost-effective than that based on molecular techniques. Designing species-specific primer for each species and current state-of-the-art molecular diagnostic tools is costly and complex, particularly in limited resource settings. Although we emphasize on DNA-based diagnostic, several issues are still relevant, and understanding financial barrier and solutions like the broadest access must be considered. Therefore, the current study was aimed at characterizing two common nematode species, *O. columbianum* and *H. contortus*, isolated from the intestine of the indigenous goats based on morphometric and economic PCR protocol. In our study, for the first time, we characterized adult *O. columbianum* and *H. contortus* in Sylhet, Bangladesh, combined with morphometry and sequence analysis. Findings of the study may contribute to a better understanding of the morphological traits and identification of these nematodes, especially in the Indian subcontinent where GINs have a worrying role in small ruminant farming.

## 2. Materials and Methods

### 2.1. Specimens and Morphological Analysis

Naturally infected adult nematodes were obtained from the abomasum of the large intestine of Black Bengal goats (*Capra hircus*) from local abattoirs in Sylhet, Bangladesh (geographically located at 24.89°N 91.88°E), in January 2020. The Black Bengal goat is an indigenous goat breed and found all over Bangladesh. Collected nematodes were washed in 0.9% saline, fixed into 70% ethanol and 10% neutral-buffered formalin, and brought to the Department of Parasitology, Chungbuk National University, Republic of Korea, for further studies. Parasite materials (PRB001197 and PRB001198) used in this study were stored in the International Parasite Resource Bank (iPRB), Republic of Korea. For morphological observation, the worms were placed in glycerine alcohol solution (90 ml 70% ethanol and 10 ml glacial glycerine) for 24 hr until they become transparent, then mounted with glycerin jelly (10 g gelatin, 500 ml glycerine, 10 g phenol, and 60 ml distilled water). Observations and measurements were conducted under a light microscope (Olympus BX-53, Tokyo, Japan) with an ocular micrometer. The following measurements (in millimeter) were taken: body length, body width, esophageal length, esophageal width, length of spicule, length of gubernaculum, distance from the vulva to posterior end, and length of tail. Buccal cavity and vulvar flap morphology were also used to make species identification.

### 2.2. DNA Extraction

DNA extraction was done from ethanol-preserved samples following a previous protocol [12]. Collected adult worms were washed 3 times in PBS before DNA extraction. Total genome DNA from individual worm was extracted by using a commercial kit (DNeasy Blood and Tissue Kit, Qiagen, Hilden, Germany; Cat Nos. 69504 and 69506). Except the elution step, where distilled water was used instead of elution buffer and was repeated twice, the remaining of the DNA extraction was performed according to the manufacturer's protocol. The concentration and purity of DNA were measured (NanoDrop Spectrophotometer, Thermo Fisher Scientific Solutions Co., Ltd., Korea), and stored at –20°C until required for PCR.

### 2.3. PCR Amplification and DNA Sequencing

The rDNA region spanning ITS region (ITS1, 5.8S, and ITS2) and a region within mitochondrial cytochrome *c* oxidase subunit I (*cox*1) were amplified and sequenced by cycle sequencing. The target regions were amplified by the primer sets (Table 1) previously described by Hu et al. [13] and Jacquiet et al. [14]. The PCR reactions were performed in a Kyratec PCR Thermal Cycler (Queensland, Australia). The volume of mixture was similar for both primer pairs and was carried out in a final reaction mixture containing 25 *μ*l, including 1 *μ*l of each primer (10 pmol), 1 *μ*l of generic DNA, 6 *μ*l of 5X PCR Master Mix (ELPIS biotech, South Korea), and 16 *μ*l of distilled water. Negative control was applied in each run. PCR condition for *cox*1 was 94°C for 3 min; (94°C 45 sec; 48°C 1 min; 72°C for 1 min) × 35; and 72°C 10 min. PCR condition for ITS was 95°C for 10 min; (95°C 45 sec; 55°C 50 sec; 72°C for 50 sec) × 30; and 72°C 5 min. When amplifications did not work adequately, the annealing temperature was changed and adjusted. The PCR products were run on a 1.5% agarose gel and visualized using a UV transilluminator. DNA sequencing was performed by a company (Cosmogenetech, Daejeon, Korea). Cycle sequencing was performed using a BigDye terminator kit (version 3.1, Applied Biosystems, Foster City, California, USA). The reaction products were directly sequenced using a DNA sequencer (ABI3730XL, Applied Biosystems).

### 2.4. Sequencing and Phylogenetic Analysis

The obtained sequences were assembled with Geneious program 9.0 (Biometer, Auckland, New Zealand) [15]. Sequences were aligned using ClustalW multiple alignment implanted MEGA7 [16, 17]. Both alignments were trimmed to the length of the shortest sequence. Sequencing analysis was carried out by BLAST algorithms and databases from the National Center for Biotechnology Information database. Phylogenetic trees were constructed based on ITS region of the newly obtained sequences, and selected reference sequences available in GenBank, using maximum likelihood (ML) algorithms with bootstrap values calculated using 1000 replicates. The multiple alignments were performed with the program Muscle [18], and substitution model (T92+G) was chosen according to the Modeltest using MEGA7. To describe the best substitution patterns, the lowest BIC (Bayesian Information Criterion) scores were considered.

## 3. Results and Discussion

In this study, adult worm specimens were precisely characterized by morphological features and DNA analysis, based on both nuclear ITS and *cox*1 gene. We choose these markers due to their high interspecific levels of variability and the availability of universal primers.

### 3.1. *Oesophagostomum* Columbianum (Curtice, 1890) Stossich 1899

**Figure 1:**
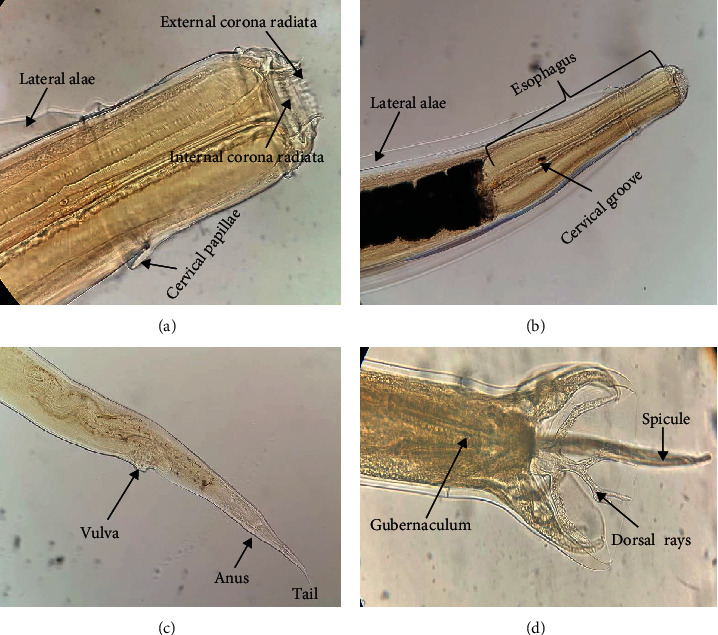
*Oesophagostomum columbianum*: (a, b) anterior end showing corona radiata and esophagus; (c) posterior end of female showing vulva and anus; (d) bursa of male showing dorsal rays and spicules.

Description of worms was based on 3 female and 2 fully mature worms as whole-mounted specimens (Figure 1; Table 2). The descriptions are as follows: medium-sized worms with sexual dimorphism, males being smaller and thinner than females; body straight, tapering at both ends with a transversally striated cuticle; anterior end curved dorsally into a hook; well-developed lateral alae extending nearly the entire length of the body, and surrounded by a well-demarcated cylindrical and sub-globular oral collar; mouth has ring-like projection leading to a small buccal capsule, surrounded by leaf-like structures which constitute the leaf-crown; buccal capsule surrounded by external corona radiata and the internal corona radiata (Figure 1(a)); internal corona radiata present with numerous elements; cephalic vesicle located just before the middle of the esophagus and demarcated by the cervical groove (Figure 1(b)); well-developed esophagus, club-shaped, shrinks immediately behind the esophageal duct, and dilates gradually to the posterior end.

The following are descriptions for the male: total body length 12.3-13.1 [12.7]; maximum width 0.29-0.37 [0.33]; length of esophagus 0.76-0.78 [0.77]; maximum width of esophagus 0.09-0.11 [0.10]; length of buccal capsule 0.05, depth of mouth capsule 0.09. Copulatory bursa symmetrical, bell-shaped, with ventrolateral lobes; dorsal ray broad at origin, 0.15-0.19 [0.17] in length, arising from a common dorsal trunk and divided into two terminal branches, each of which gives a short lateral stem; ventral rays end quite near to the border of the lateral lobe; spicules equal, slender, alate with blunt tips, 0.73-0.79 [0.75] long; gubernaculum elongate, margins irregular, length 0.09-0.13 [0.11] (Figure 1(d)).

The following are descriptions for the female: total body length 15.6-16.8 [16.2]; maximum width 0.30-0.38 [0.34]; length of esophagus 0.77-0.81 [0.79], width of the esophagus 0.1-0.12 [0.11]; length of mouth capsule 0.05, depth of mouth capsule 0.10; tail straight and conical; distance of anus from posterior end of body 0.31-0.33 [0.32]; vulva elliptical, slightly pronounced, anterior to the anus and opens 0.71-0.83 [0.77] from posterior end (Figure 1(c)).

Our specimens were identified as *O. columbianum* as well as differentiated from the other *Oesophagostomum* species considering host and characteristics of buccal capsule with other cephalic and cervical structures, spicule, and gubernacular length and position of the vulva and anus. The morphology and morphometry of the present *Oesophagostomum* specimens were identical to those of *O. columbianum* documented previously [19–23]. According to Railliet and Henry [20], one or more of the following characteristics such as the number of elements in corona radiata, position of cervical papillae, and structure of male bursa seem to be the most appropriate generic features of *Oesophagostomum*. In our specimens, cervical papillae were observed anterior to esophageal expansion which is consistent with the description. This species was discussed in some detail by Goodey [22] where he stated that the structure of anterior parts and spicule length of this genus are so crucial to the systematics and are the key to species differentiation. The male spicule length of *O. columbianum* is varying from 0.75 to 0.80, and cephalic vesicle is not distinct. Other *Oesophagostomum* species of small ruminants like *O. asperum* has well-defined cephalic vesicles and somewhat longer spicules. Furthermore, Goodey [22] and Zhao et al. [24] reported well-developed lateral alae in *O. columbianum*, while other *Oesophagostomum* of small ruminants has no lateral alae. Therefore, all of these criteria evidently indicate our specimen as *O. columbianum*.

Due to limited resources, designing and applying specific primers for individual helminth species are difficult for researchers from poor-resource countries. To address this issue, we choose universal primer set instead of species-specific primers, to validate the capacity of universal primers and to identify nematodes. rDNA and *cox*1 markers have also been used to identify successfully nematode species in *Strongyloides* [11]. In our study, the *cox*1 sequence identities of *Oesophagostomum* species ranged from 96.2% to 96.9%, when compared with reference sequences from GenBank databases using BLAST search (http://www.ncbi.nlm.nih.gov/BLAST/). Hebert et al. [25] described that the percentage of sequence divergences at the *cox*1 gene for the same generic species of nematodes estimated falling in a particular range is 4.8%. The sequence of Bangladesh-origin specimen is most closely related to but distinct from Chinese isolate *O. columbianum* from sheep (GenBank No. KC715827), indicating that the parasite has acquired genetic changes after entering into the new host (goat) and new location (Bangladesh). In Bangladesh, various host species such as sheep, goats, cattle, and buffaloes share similar grazing fields; thus, breaking down the host barrier and moving parasites from one host to another are a common occurrence [26]. While several studies mentioned that universal primer-based *cox*1 gene sequences have limitation to identify nematode in species-level previously, most of the studies have been conducted based on the rDNA, particularly from the ITS region [24, 27]. Sequence analysis of this genetic marker provides an intent and specific means of species identification. In this study, the ITS (ITS1-5.8S-ITS2) sequences of *Oesophagostomum* species showed a similarity of 98.4% to 98.8% with previously published *O. columbianum* sequences and showed to be closer to Chinese isolate *O. columbianum* from sheep (GenBank No. JX188470). These differences may be associated with the geographical origins and animal husbandry system. Application of additional genetic markers may be a good option to verify the species identification; however, it should be verified by morphological observation. Also, very few sequences targeting ITS1-5.8S-ITS2 region of rDNA are currently available in the NCBI database.

**Figure 2:**
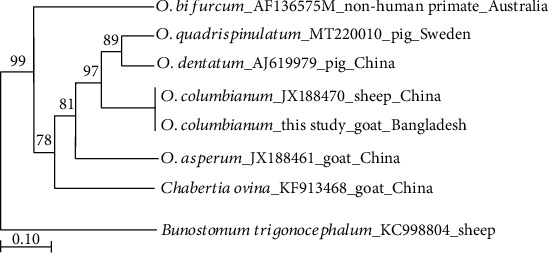
Phylogenetic relationships of present specimen (*O. columbianum*) with other members of Oesophagostominae reconstructed by ML method based on the ITS sequences. Bootstrap values are shown above branches. The scale bar represents 0.10% divergence.

To determine the taxonomic positions, a genetic tree of *O. columbianum* among other members of the genus *Oesophagostomum* was built using the ML method (Figure 2). The tree was reconstructed utilizing ITS sequences and *Bunostomum trigonocephalum* (GenBank No. KC998804) was used as the outgroup. The phylogenetic data revealed that *O. columbianum* isolated in Sylhet, Bangladesh, was closely related to the Chinese isolate. Because of commercial relationship between Bangladesh and China, as well as neighboring countries (India, Myanmar, and Pakistan), it might be possible that the origin of *O. columbianum* had been found in China. On the tree, our specimen is positioned in the sister clade of two nodule worms, *O. dentatum* and *O. quadrispinulatum*. The DNA sequence results reported herein were consistent with previous studies of Dorris et al., Zhao et al., and Newton et al. [11, 24, 28].

### 3.2. *Haemonchus contortus* (Rudolphi, 1803) Cobb, 1898

**Figure 3:**
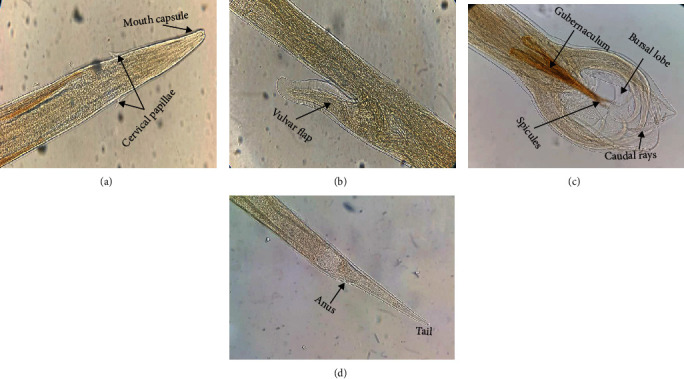
*Haemonchus contortus*: (a) anterior end showing cervical papillae; (b) knobbed vulvar flab of female; (c) bursa of male showing dorsal rays and spicules; (d) posterior end of female showing the anus and tail.

Description of worms was based on 3 female and 2 fully mature male as whole-mounted specimens (Figure 3; Table 3). Filiform- (cylindrical) shaped worms are relatively small-sized, tapering towards the anterior end in males and both ends in females. Posterior tip of females showed a “barber pole,” while males appeared to expand in a copulatory bursa (Figure 3(c)). No morphological differences were observed in the anterior structures of male and female; both had a small buccal capsule and a long esophagus.

The following are descriptions for the male: total body length 18.8-20.4 [19.6]; maximum width 0.31-0.43 [0.36]; length of esophagus 1.55-1.67 [1.61]; maximum width of esophagus 0.11-0.15 [0.13]; shoulder region showed a pair of wedge-shaped cervical papillae (Figure 3(a)); distance of cervical papillae from anterior end of body 0.37-0.45 [0.41]. Copulatory bursa asymmetrical with two distinct lobes; lateral lobe containing an inverted Y-shaped dorsal ray (bifurcated); lateral rays arise from a common trunk, and ventral rays are fused proximally and separated dorsally; paired spicules, equal, 0.40-0.46 [0.43] long; gubernaculum elongate, length 0.19-0.23 [0.21].

The following are descriptions for the female: total body length 26.8-27.4 [27.1]; maximum width 0.39-0.47 [0.43]; length of esophagus 1.88-1.96 [1.92], width of the esophagus 0.09-0.15 [0.12]; distance of cervical papillae from anterior end of body 0.45-0.51 [0.48]; tail thin, straight, and pointed (Figure 3(d)); distance of the anus from posterior end of the body 0.46-0.54 [0.50]; vulva located in the posterior third of the body and covered by a prominent knob-shaped vulvar flap (Figure 3(b)) and opens 4.30-4.54 [4.42] from posterior end.

The morphological observation and morphometric features of the present specimens were consistent with the description of the genus *Haemonchus* and were identical to those of *H. contortus* documented previously [5, 21, 29–31]. *H. contortus* is one of the nematodes of both domestic and wild mammals, most commonly encountered all over the world, and variation in the different measurements can, therefore, be expected. According to the characteristics of the gastrointestinal Trichostrongylidae described by Daskalov [31] and Santiago [5], vulvar flap of female, bifurcated dorsal rays of male, and the egg size can be considered appropriate parameters in the identification and distinguishing of genus *Haemonchus* from other GINs. However, intragenus morphological differentiation between various *Haemonchus* species is arbitrary; many variations and combination of characteristics make it difficult in precise identification. Though several studies have showed the differences between *H. placei* and *H. contortus*, there is still some argument concerning their identities. Taylor et al. [32] stated that *H. contortus* and *H. placei* are the single species and *H. contortus* with only strain adaptation for domestic ruminant. However, this statement was proven inconsistent by do Amarante [33] where he mentioned substantial morphological, biological, and genetic evidence of the existence of both species. Also, Lichtenfels et al. [5] distinguished *H. contortus* from *H. similis* and *H. placei* based on spicule structure and length and vulvar structure and tail length. According to his description, the mean spicule length for *H. contortus* is around 0.38-0.42, whereas the average spicule length for *H. placei* and *H. similis* is 4.3-5.1 and 3.0-3.8, respectively. The mean tail length for *H. contortus* is around 0.25-0.53, whereas the average spicule length for *H. placei* and *H. similis* is 0.37-0.72 and 0.13-0.27, respectively. The spicule length (0.40-0.46) and tail length (0.46-0.54) of our specimen were consistent with the distinctive characteristics of *H. contortus* given by Lichtenfels et al. [5].

**Figure 4:**
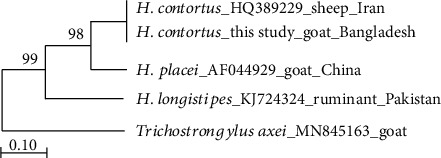
Phylogenetic relationships of present specimen (*H. contortus*) with other members of Haemonchidae reconstructed by the ML method based on the ITS sequences and numbers at the branch nodes indicate percentage bootstrap support for 1000 replicates.

Molecular identification of *H. contortus* was examined by sequence differences in the *cox*1 and ITS regions with other *Haemonchus* species including *H. placei* and *H. similis*. The *cox*1 sequence identities of Bangladesh-origin *Haemonchus* species ranged from 96.8% to 97.3%, showing the highest homology to the *H. contortus* isolate from goat in Pakistan (GenBank No. KJ724377). On the other hand, within ITS1-5.8S-ITS2 sequences, identities ranged from 97.7% to 99%, showing the highest homology to the *H. contortus* isolate from sheep in Iran (GenBank No. HQ389229). Meshgi et al. [34] revealed the similar observation and reported no molecular difference between *H*. *contortus* from sheep and goat isolates. To determine the taxonomic positions of the present specimen, a phylogenetic tree among members of the genus *Haemonchus* was constructed using ML, based on the ITS sequences (Figure 4), with *Trichostrongylus axei* (GenBank No. MN845163) as the outgroup. The phylogenetic data revealed that the Bangladesh origin *H. contortus* showed similarity with Iranian isolate. In such a scenario, the reason is the introduction of the parasite population by imported animals of the same origin as there are evidences of direct animal movement between Bangladesh and Iran through the neighboring countries, especially during the period of Islamic religious festival (Eid-ul-Adha). In addition, there is no strong geographical barrier of Bangladesh between neighboring countries and free animal movement and border crossing for legal and illegal trading from neighboring countries are widespread. Dey et al. [26] and Troell et al. [35] reported similar observations where Malaysian isolates showed close association with American isolates, and Greek isolates overlapped with Australian isolates. On the tree, our specimen was grouped in one clade sister to *H. placei* but separated from other *Haemonchus* species. This result is consistent with the findings described by Akkari et al. [7], Troell et al. [35], and Gasser et al. [36].

In our study, we identified *O. columbianum* and *H. contortus* based on morphological properties, morphometry, and molecular analysis, which parasitized indigenous goat. Since precise identification of parasites at appropriate taxonomic levels is essential for the control of parasitosis, the present findings have significant implications for studying epidemiology and designing control strategies. Both species are widespread and have been documented from different parts of the Indian subcontinent time to time, including Meghalaya [23, 37]. The geographical location of that areas is close to our study area, which thereby forms a new locality record for these species. The present findings describing morphological differences along with the genetic data provided here provide solid evidence for a distinct species of parasitizing goat *Oesophagostomum* and *Haemonchus*. Although there has been no zoonotic transmission report of these nematodes till now, further investigation is needed to determine the probability of the risk. There are a few limitations to our study. First, we collected adult worm from the slaughtered goats at abattoirs. For this reason, we were unable to determine the prevalence of these parasites in the study areas. Secondly, we did not collect faecal samples and eggs were not observed during morphological examination of adult females, which make us unable to describe and measure the egg size of the parasites. Nevertheless, there is a need for additional epidemiological surveys across different geographical settings to unravel the detailed morphology.

## 4. Conclusions

We identified *Oesophagostomum columbianum* and *Haemonchus contortus* from the goat in Bangladesh. To our knowledge, for the first time, we obtained DNA sequences of *O. columbianum* in Bangladesh and *H. contortus* in Sylhet, Bangladesh. Findings of our study indicated a high specificity and sensitivity of ITS region in comparison to the *cox*1 gene for identifying nematodes. Also, the universal primers, if protocol is accurately designed, could give precise results and could be an economic option for the researchers from poor-resource settings. However, to describe genetic diversity in more detail, additional observation of specimens from different geographic settings and hosts will be helpful. Findings of our study might have a significant implication for the epidemiology, taxonomy, and population dynamics, as well as for the management and control of these nematodes.

## Figures and Tables

**Table 1:** Primer sets used, with nucleotide sequence, target region, species, and size of amplicon.

Primers	Target region	Species	Amplicon size (bp)
NC5: 5′-gtaggtgaacctgcggaaggatcatt-3′ NC2: 5′-ttagtttcttttcctccgct-3′	ITS (rDNA)	*O. columbianum*	782
		*H. contortus*	789
JB3: 5′-ttttttgggcatcctgaggtttat-3′ JB4.5: 5′-taaagaaagaacataatgaaaatg-3′	*cox*1 (mtDNA)	*O. columbianum*	397
		*H. contortus*	383

**Table 2:** Comparative measurement (in mm) of present *Oesophagostomum columbianum* isolated and those previously recorded.

Body parts	Present specimen	Goodey (1924)	Ransom (1911)	Soota (1981)
Male				
Body length	12.3-13.1	12-14	12-16	9-14
Body width	0.29-0.37	0.23-0.40	—	—
Length of the esophagus	0.76-0.78	—	—	0.6-0.8
Length of spicules	0.73-0.79	0.75-0.80	0.75-0.85	0.7-0.8
Length of the gubernaculum	0.09-0.13	0.10-0.15	0.1	—
Female				
Body length	15.6-16.8	15-18	14-18	12-16
Body width	0.30-0.38	0.30-0.5	—	—
Length of the esophagus	0.77-0.81	—	—	0.7-0.8
Distance of the vulva from posterior end of the body	0.71-0.83	0.75-0.80	0.9-1.0	0.71-1.03
Distance of the anus from posterior end of the body	0.31-0.33	0.5-0.6	0.3	0.31-0.42

**Table 3:** Comparative measurement (in mm) of present *Haemonchus contortus* isolated and those previously recorded.

Body parts	Present specimen	Santiago (1968)	Ransom (1911)	Lichtenfels et al., (1994)
Male				
Body length	18.8-20.4	14-17	10-20	11.0-17.0
Maximum thickness	0.31-0.43	0.199-0.265	0.40	
Length of the esophagus	1.55-1.67	1.444-1.743	1.5	1.09-1.55
Cervical papillae	0.37-0.45	—	—	0.27-0.46
Length of spicules	0.40-0.46	0.398-0.448	0.30-0.50	0.38-0.47
Length of gubernaculum	0.19-0.23	0.199-0.349	0.20	0.19-0.25
Female				
Body length	26.8-27.4	20-27	18-30	14.8-27.2
Body thickness	0.39-0.47	0.215-0.332	0.50	—
Length of the esophagus	1.88-1.96	1.162-1.662	—	1.15-1.66
Cervical papillae	0.45-0.51	—	—	0.24-0.48
Distance of the vulva from posterior end of the body	4.30-4.54	3.81-5.31	3-4.5	3.01-4.90
Distance of the anus from posterior end of the body	0.46-0.54	0.415-0.513	0.40-0.63	0.25-0.53

## Data Availability

Genomic DNA were stored in International Parasite Resource Bank (iPRB) and available from the corresponding author on reasonable request. The sequences obtained from Bangladesh isolates of *Oesophagostomum columbianum* and *Haemonchus contortus* were deposited in GenBank (accession numbers MT653093 and MT645506).

## Abbreviations

GINs: Gastrointestinal nematodes
mtDNA: Mitochondrial DNA
rDNS: Ribosomal DNA
*cox*1: Cytochrome oxidase *c* subunit 1
ITS: Internal transcribed spacer (ITS1-5.8S-ITS2)
PCR: Polymerase chain reaction
T92+G: “Tamura 1992 + Gamma” mathematical method
ML: Maximum likelihood
mm: Millimeter.

## References

[1] Whitley N. C., Oh S. H., Lee S. J. (2014). Impact of integrated gastrointestinal nematode management training for U.S. goat and sheep producers. *Veterinary Parasitology*.

[2] Tariq K. A., Chishti M. Z., Ahmad F. (2010). Gastro-intestinal nematode infections in goats relative to season, host sex and age from the Kashmir valley, India. *Journal of helminthology*.

[3] McCarthy J., Moore T. A. (2000). Emerging helminth zoonoses. *International Journal for Parasitology*.

[4] Biffa D., Jobre Y., Chakka H. (2006). Ovine helminthosis, a major health constraint to productivity of sheep in Ethiopia. *Animal Health Research Reviews*.

[5] Lichtenfels J. R., Pilitt P. A., Hoberg E. P. (1994). New morphological characters for identifying individual specimens of Haemonchus spp. (Nematoda: Trichostrongyloidea) and a key to species in ruminants of North America. *The Journal of Parasitology*.

[6] Nath T. C., Bhuiyan M. J. U., al Mamun M. (2014). Common infectious diseases of goats in Chittagong district of Bangladesh. *International Journal of Scientific Research in Agricultural Sciences*.

[7] Akkari H., Jebali J., Gharbi M., Mhadhbi M., Awadi S., Darghouth M. A. (2013). Epidemiological study of sympatric *Haemonchus* species and genetic characterization of *Haemonchus contortus* in domestic ruminants in Tunisia. *Veterinary Parasitology*.

[8] Almeida J. L. (1933). Note sur les espèces du genre Haemonchus Cobb, 1898 (Nematoda- Trichostrongyloidea). *Comptes Rendues des Séances de la Société de Biologie et de ses Filiales*.

[9] Mazid M. A., Bhattacharjee J., Begum N., Rahman M. H. (2008). Helminth parasites of the digestive system of sheep in Mymensingh, Bangladesh. *Bangladesh Journal of Veterinary Medicine*.

[10] Mohanta U. K., Anisuzzaman T., Farjana P., Das M., Majumder S., Mondal M. M. H. (2007). Prevalence, population dynamics and pathological effects of intestinal helminths in Black Bengal goats. *Bangladesh Journal of Veterinary Medicine*.

[11] Dorris M., De Ley P., Blaxter M. L. (1999). Molecular analysis of nematode diversity and the evolution of parasitism. *Parasitology Today*.

[12] Eom K. S., Park H. S., Lee D. M. (2019). Identity of *Spirometra theileri* from a leopard (*Panthera pardus*) and spotted hyena (*Crocuta crocuta*) in Tanzania. *The Korean Journal of Parasitology*.

[13] Hu M., Chilton N. B., Gasser R. B. (2002). The mitochondrial genomes of the human hookworms, *Ancylostoma duodenale and Necator americanus* (Nematoda: Secernentea). *International journal for parasitology*.

[14] Jacquiet P., Humbert J. F., Comes A. M., Cabaret J., Thiam A., Cheikh D. (1995). Ecological, morphological and genetic characterization of sympatric Haemonchus spp. parasites of domestic ruminants in Mauritania. *Parasitology*.

[15] Kearse M., Moir R., Wilson A. (2012). Geneious Basic: an integrated and extendable desktop software platform for the organization and analysis of sequence data. *Bioinformatics*.

[16] Kumar S., Stecher G., Tamura K. (2016). MEGA7: molecular evolutionary genetics analysis version 7.0 for bigger datasets. *Molecular Biology and Evolution*.

[17] Thompson J. D., Higgins D. G., Gibson T. J. (2004). CLUSTAL W: improving the sensitivity of progressive multiple sequence alignment through sequence weighting, position-specific gap penalties and weight matrix choice. *Nucleic Acids Research*.

[18] Edgar R. C. (2004). MUSCLE: multiple sequence alignment with high accuracy and high throughput. *Nucleic Acids Research*.

[19] Railliet A. (1909). Une seconde espe’ce d’oesophagostome parasite de l’homme. *Bulletin de la Société de Pathologique Exotique*.

[20] Railliet A., Henry A. (1913). Surles Oesophagostomietts des Ruminants. *Bulletin de la Société de pathologie exotique*.

[21] Ransom B. H. (1911). *The nematodes parasitic in the alimentary tract of cattle, sheep and other ruminants*.

[22] Goodey T. (1924). Oesophagostome of goats, sheep, and cattle. *Journal of Helminthology*.

[23] Soota T. D. (1981). On some nematodes from the unnamed collection of the zoological survey of India, along with the description of a new species. *Records of the Zoological Survey of India*.

[24] Zhao G. H., Hu B., Cheng W. Y. (2013). The complete mitochondrial genomes of *Oesophagostomum asperum* and *Oesophagostomum columbianum* in small ruminants. *Infection, Genetics and Evolution*.

[25] Hebert P., Cywinska A., Ball S., deWaard J. R. (2003). Biological identifications through DNA barcodes. *Proceedings of the Royal Society of London. Series B: Biological Sciences*.

[26] Dey A. R., Zhang Z., Begum N., Alim M. A., Hu M., Alam M. Z. (2019). Genetic diversity patterns of *Haemonchus contortus* isolated from sheep and goats in Bangladesh. *Infection, Genetics and Evolution*.

[27] Nabavi R., De Waal T., Conneely B., McCarthy E., Good B., Shayan P. (2014). Comparison of internal transcribed spacers and intergenic spacer regions of five common Iranian sheep bursate nematodes. *Iranian Journal of Parasitology*.

[28] Newton L. A., Chilton N. B., Beveridge I., Gasser R. B. (1998). Systematic relationships of some members of the genera Oesophagostomum and Chabertia (Nematoda: Chabertiidae) based on ribosomal DNA sequence data. *International journal for parasitology*.

[29] Rudolphi C. A. (1803). Neue Beobachtungen über die Eingeweidewürmer. *Archiv für Zoologie und Zootomie*.

[30] Santiago M. A. M. (1968). *Haemonchus Cobb,1898 (Nematoda: Trichostrongylidae). Contribuição ao estudo da morfologia, biologia e distribuição geográfica das espécies parasitas de ovinos e bovinos no Rio Grande do Sul*.

[31] Daskalov P. (1963). Morphological differences in females of Haemonchus contortus and H. placet. *Izvestiya na Tsentralnata Khelmintologichna Laboratoriya*.

[32] Taylor M. A., Coop R. L., Wall R. L. (2010). *Parasitologia Veterinária*.

[33] Amarante A. F. T. (2011). Why is it important to correctly identify Haemonchus species?. *Revista Brasileira de Parasitologia Veterinária*.

[34] Meshgi B., Jalousian F., Masih Z. (2015). Phylogenetic study of *Haemonchus* species from Iran based on morpho-molecular characterization. *Iranian Journal of Parasitology*.

[35] Troell K., Engström A., Morrison D. A., Mattsson J. G., Höglund J. (2006). Global patterns reveal strong population structure in *Haemonchus contortus*, a nematode parasite of domesticated ruminants. *International Journal for Parasitology*.

[36] Gasser R. B., Newton S. E. (2000). Genomic and genetic research on bursate nematodes: significance, implications and prospects. *International Journal for Parasitology*.

[37] Khera S. (1954). Nematode parasites of some Indian vertebrates. *Indian Journal of Helminthology*.

